# Peripheral Osteoma of the Mandibular Notch: Report of a Case

**DOI:** 10.5812/iranjradiol.3734

**Published:** 2013-05-20

**Authors:** Toshinori Iwai, Toshiharu Izumi, Junichi Baba, Jiro Maegawa, Kenji Mitsudo, Iwai Tohnai

**Affiliations:** 1 Department of Oral and Maxillofacial Surgery, Yokohama City University Graduate School of Medicine, , Yokohama, Kanagawa, Japan; 2 Department of Radiology, Yokohama City University Hospital, Yokohama, Kanagawa, Japan; 3 Department of Plastic and Reconstructive Surgery, Yokohama City University Hospital, Yokohama, Kanagawa, Japan

**Keywords:** Tomography, X-Ray Computed, Osteoma, Mandible

## Abstract

Osteoma is a benign, slow-growing osteogenic tumor that sometimes arises from the craniomaxillofacial region, such as the sinus, temporal or jaw bones. Osteoma consists of compact or cancellous bone that may be peripheral, central or extraskeletal type. Peripheral osteoma arises from the periosteum and is commonly a unilateral, pedunculated mushroom-like mass. Peripheral osteoma of the mandible is relatively uncommon, and peripheral osteoma of the mandibular notch is extremely rare, although many cases arise from the mandibular body, angle, condyle, or coronoid process. We report here an unusual peripheral osteoma of the mandibular notch in a 78-year-old nonsyndromic female.

## 1. Introduction

Osteoma is a benign, slow-growing osteogenic tumor that sometimes arises from the craniomaxillofacial region, such as the sinus, temporal bone, or jaw bone (1-3). Osteoma is typically a solitary lesion, but patients with an osteoma should be evaluated for Gardner’s syndrome, which is characterized by multiple osteomas, gastrointestinal polyps, skin and soft tissue tumors and multiple impacted or supernumerary teeth (4). Osteoma consists of compact or cancellous bone (5) and can be of a peripheral, central, or extraskeletal type (2, 6). Peripheral osteoma arises from the periosteum and is commonly a unilateral, pedunculated mushroom-like mass (2, 3, 7). Peripheral osteoma of the mandible is relatively uncommon (2, 8) and peripheral osteoma of the mandibular notch is extremely rare (5, 8, 9), although many cases arise from the mandibular body, angle, condyle or coronoid process (1, 3, 6, 10). The incidence of the mandibular notch peripheral osteoma is 1.6% among mandibular peripheral osteomas (6). To our knowledge, only three cases have been reported in the English literature (5, 8, 9). We report here an unusual peripheral osteoma of the mandibular notch.

## 2. Case Presentation

A 78-year-old nonsyndromic female with a tongue ulcer was referred to our department. Biopsy was done and the pathological diagnosis was squamous cell carcinoma of the tongue. In the panoramic radiograph, a radiopaque lesion was located between the coronoid process and the condyle. A 64-detector spiral computed tomography (CT) scanner (Aquilion 64; Toshiba Medical, Tokyo, Japan) was used. CT showed a 36 × 35 × 30 mm sized lesion arising from the right mandibular notch (Figure 1). Three-dimensional CT revealed that the lesion was growing medially, laterally, and upward from the lingual aspect of the mandibular notch, avoiding the zygomatic arch (Figure 2).

**Figure:**
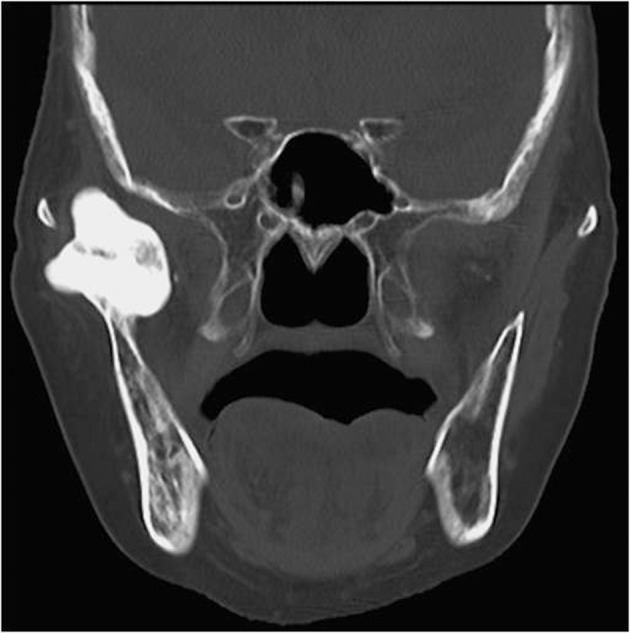
Figure 1. Coronal CT image. A 36 × 35 × 30 mm sized lesion is seen that arises from the right mandibular notch.

**Figure:**
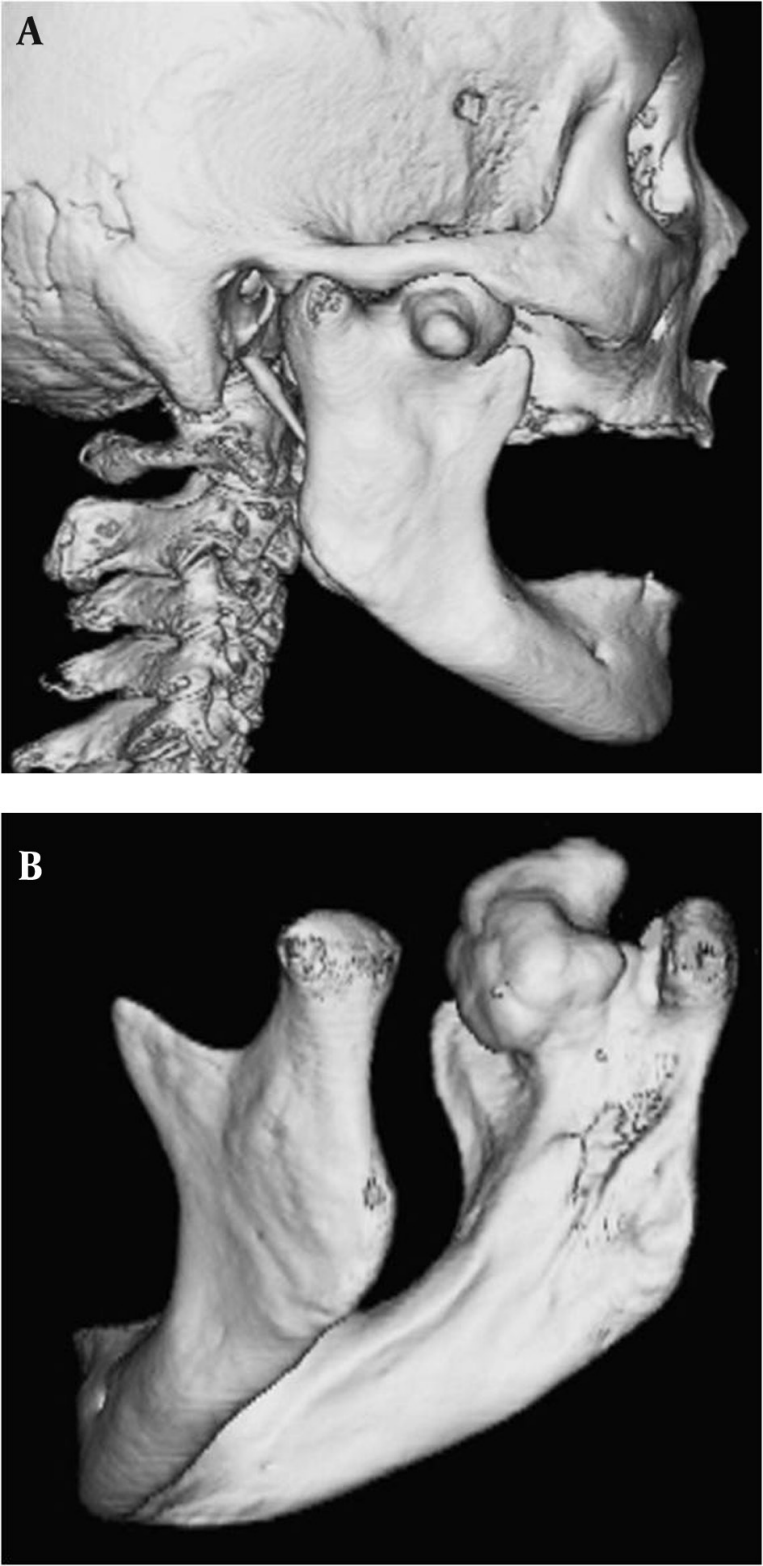
Figure 2. A. Lateral and B. Medial view of three-dimensional CT image shows a bony mass arising from the mandibular notch.

Radiological diagnosis was peripheral osteoma. There was no trismus, temporomandibular joint dysfunction, deviation of the mandible, facial asymmetry, or neurological abnormal finding in the mental region. The patient had no history of trauma or infection in the right mandibular region. As the patient declined surgery for both tongue cancer and peripheral osteoma of the mandibular notch, superselective intra-arterial chemoradiotherapy for tongue cancer was performed for organ preservation and the osteoma was observed. The patient received daily concurrent chemoradiotherapy. The intra-arterial chemotherapy was performed via bilateral superficial temporal arteries with docetaxel and cisplatin at a dose of 15 mg/m^2^/week and 5 mg/m^2^/day, respectively. External beam irradiation of the tongue cancer was performed 5 times per week at 1.8 Gy per fraction, for a total of 59.4 Gy. Because the treatment effect of tongue cancer was complete response, the patient had a high quality of life without dysphagia. There was no evidence of recurrence or metastasis 5 years after chemoradiotherapy, and the size of the mandibular notch osteoma was unchanged on clinical and radiological examination.

## 3. Discussion

The precise etiology of osteoma is unknown, although developmental anomaly, true neoplasm, reactive lesion triggered by trauma, infection, and muscle traction have been proposed (1-3). Kaplan et al. (1) suggested that many peripheral osteomas may be reactive lesions caused by trauma or muscle traction rather than neoplasm, because many peripheral osteomas are located on the lower border or buccal aspect of the mandible. None of these etiological factors could be associated with the present case arising from the medial aspect of the mandibular notch.

As osteoma may be clinically silent for years without symptoms, it is usually diagnosed when it becomes enlarged or is incidentally discovered by radiological examination such as panoramic radiography or CT (8). CT (with three-dimensional reconstruction) is considered the best imaging modality both to identify the location and extent of the lesion (2, 3, 5, 8) and to determine the surgical approach (7, 8). Treatment includes surgery or observation. Small, nonprogressing, asymptomatic, solitary osteomas may reasonably be observed with periodic clinical and radiological examination, although surgery should be considered for peripheral osteomas that are large, deforming, progressive, or associated with other symptoms (8).

We reviewed peripheral osteomas of the mandibular notch, including the present case (Table 1). There were two males and two females, with a mean age of 59 years. Three were found on the right and one on the left side. Two cases were on the medial side of the mandibular notch, one case was in the middle, and one case was on the lateral side. The mean major and minor axes were 33.3 mm and 24.7 mm, respectively. In the previous three cases, the osteomas were below the zygomatic arch, whereas the present case grew laterally and upward, avoiding it. One patient had diffuse maxillary pain, one had buccal swelling, and two had no symptoms. Only one patient reported by Bessho (9) had previous trauma in the right buccal region. Treatment for the patient with maxillary pain was not documented (5). Although the patient with buccal swelling underwent surgery, the two patients without symptoms including the present case were observed, with follow-up periods of 1 to 5 years with regular clinical and radiological examinations, and there were no changes in tumor size during observation.

**Table 1.** Summary of the reported cases of peripheral osteoma of the mandibular notch

No.	Source	Age/Sex	Side	Location	Size (mm)	Symptom	Treatment	Recurrence or growth
1	Bessho et al. (9)	26/M	R	Lateral	34x29x17	Buccal swelling	Surgery	NA
2	Schulze (5)	73/F	L	Medial	30x30x27	Maxillary pain	NA	NA
3	Sekerci et al. (8)	59/M	R	Middle	NA	-	Follow-up	-
4	Present case	78/F	R	Medial	36x35x30	-	-	-

Abbreviations: NA, Not Available
